# Outcome evaluation by patient reported outcome measures in stroke clinical practice (EPOS) protocol for a prospective observation and implementation study

**DOI:** 10.1186/s42466-019-0034-0

**Published:** 2019-11-01

**Authors:** D. Leander Rimmele, Lisa Lebherz, Marc Frese, Hannes Appelbohm, Hans-Jürgen Bartz, Levente Kriston, Christian Gerloff, Martin Härter, Götz Thomalla

**Affiliations:** 1 Department of Neurology, University Medical Centre Hamburg-Eppendorf, Martinistr. 52, 20246 Hamburg, Germany; 2 Department of Medical Psychology, University Medical Centre Hamburg-Eppendorf, Martinistr. 52, 20246 Hamburg, Germany; 3 Office for Quality Management and Clinical Process Management, University Medical Centre Hamburg-Eppendorf, Martinistr. 52, 20246 Hamburg, Germany

**Keywords:** Stroke care, Patient-reported outcome measures, Patient-relevant care, Long term assessment, Health-related quality of life, Process evaluation, Feasibility, ICHOM, Inpatient treatment

## Abstract

**Introduction:**

The impact of stroke-related impairment on activities of daily living may vary between patients, and can only be estimated by applying patient-reported outcome measures. The International Consortium for Health Outcome Measurement has developed a standard set of instruments that combine clinical and longitudinal patient-reported outcome measures for stroke. The present study was designed (1) to implement and evaluate the feasibility of the use of it as a consistent outcome measure in clinical routine at the stroke center of a German university hospital, (2) to characterize impairment in everyday life caused by stroke, and (3) to identify predictive factors associated with patient-relevant outcomes.

**Methods:**

We plan to enroll 1040 consecutive patients with the diagnosis of acute ischemic stroke, transient ischemic attack, or intracerebral hemorrhage in a prospective observational study. Demographics, cardiovascular risk factors, and living situation are assessed at inpatient surveillance. At 90 days and 12 months after inclusion, follow-up assessments take place including the Patient-reported Outcomes Measurement Information System 10-Question Short Form (PROMIS-10), the Patient- Health Questionnaire-4, and the simplified modified Ranking Scale questionnaire. The acceptance and feasibility (1) will be assessed by a process evaluation through qualitative semi-structured interviews with clinical staff and patients and quantitative analyses of the data quality evaluating practicability, acceptance, adoption, and fidelity to protocol. The primary outcome of objective 2 and 3 is health-related quality of life measured with the PROMIS-10. Additional outcomes are depressive and anxiety symptoms and patient participation in their social roles. Patient-reported outcomes will be assessed in their longitudinal course using (generalized) mixed regressions. Exploratory descriptive and inference statistical analyses will be used to find patterns of patient characteristics and predictive factors of the outcome domains.

**Perspective:**

The results will describe and further establish the evaluation of stroke patients of a stroke center by standardized PROMs in everyday life.

**Trial registration:**

The trial is registered at ClinicalTrials.gov (NCT03795948). Approval of the local ethics committee (Ethik-Kommission der Ärztekammer Hamburg) has been obtained.

## Introduction

Stroke is a major cause of death and disability worldwide. Although the mortality of stroke has decreased through improved treatment by new interventions and more efficient management strategies, the overall societal burden of stroke has further grown during the last decades [8, 17]. Three months after stroke, about 40% of stroke patients are either dead, living in a nursing home, or depending on continuous care from others [12]. Direct deficits resulting from stroke, e.g. paresis or aphasia, are easily spotted, and well characterized by clinical rating scales, which are used in clinical practice. There are, however, indirect consequences of stroke which are more difficult to detect unless they are looked for by purpose. Stroke patients are known to suffer from problems with impaired cognition, anxiety, and depression amongst others, which in turn, affect functional status, and satisfaction in daily living, and which often only become manifest in the long term in everyday life as outpatients [1, 16, 23]. These highly relevant consequences of stroke are often missed in standard assessments by physicians or in clinical scales applied by stroke researchers.

The past years have brought a movement towards a more patient-centered evaluation of quality and value in health care, summarized by the term of `value-based healthcare´ [20, 21]. In this context, the evaluation of quality of care and treatment success by patient-reported outcome measures (PROMs) play a central role [10, 24]. Retrospective studies of patients after mild ischemic, and hemorrhagic strokes have reported altered quality of life using PROMs of the National Institute of Health’s Patient-reported Outcome Measurement Information System (PROMIS), showing changes in physical, social, and mental domains [15]. To overcome the problem of the great variability of PROMs used for assessment, the International Consortium for Health Outcomes Measurements (ICHOM) has coordinated the development of a consensus Standard Set of PROMs for Stroke based on PROMIS in order to enable comparable assessment of healthcare value in stroke management across different settings [13, 14, 22]. There is, however, a lack of data on patient-reported outcomes of stroke management from routine clinical practice. Studies reporting process evaluation of implementation of PROMs in the clinical practice are also scarce, although in form of process evaluations, facilitators and barriers of such an implementation can be discovered, and the implementation process can be adjusted for a successful realization of the desired intervention or enquiry.

## Methods

### Aim of the trial

The objectives of the current study are to implement the ICHOM-Standard Set for Stroke (ICHOM-SSS) in routine stroke care, and appraise this implementation, i.e. the acceptance and feasibility of its use in clinical practice by means of a process evaluation, to systematically characterize the long-term functional status and health-related quality of life after stroke in a longitudinal setting, and to identify factors influencing outcome and course of outcome after stroke. The addressed research questions are to which extend can the ICHOM-SSS be implemented into the routine health care for stroke patients and how are acceptance, benefit, and feasibility appraised by patients and clinical staff? How is the outcome of stroke and stroke treatment in a consecutive sample and which medical and patient-related factors influence the treatment outcome?

### Study description and study design

We perform a prospective exploratory observational and implementation study with a longitudinal design. The sample consists of a consecutive cohort of patients referred to the stroke unit at the University Medical Centre Hamburg-Eppendorf for acute ischemic or hemorrhagic stroke within a 15 months period.

### Inclusion/exclusion criteria

Inclusion criteria are:

- treatment at the stroke unit of our hospital with acute ischemic stroke (AIS) [ICD10 I63], transient ischemic attack (TIA) [ICD10 G45], or intracerebral hemorrhage (ICH) [ICD10 I61]
- informed consent by the patient or legal guardian

Exclusion criteria are:

- severe disturbances of the ability to communicate, i.e. due to severe aphasia, dementia;
- insufficient knowledge of the German or English language.

### Sample size estimation

**Fig. 1:**
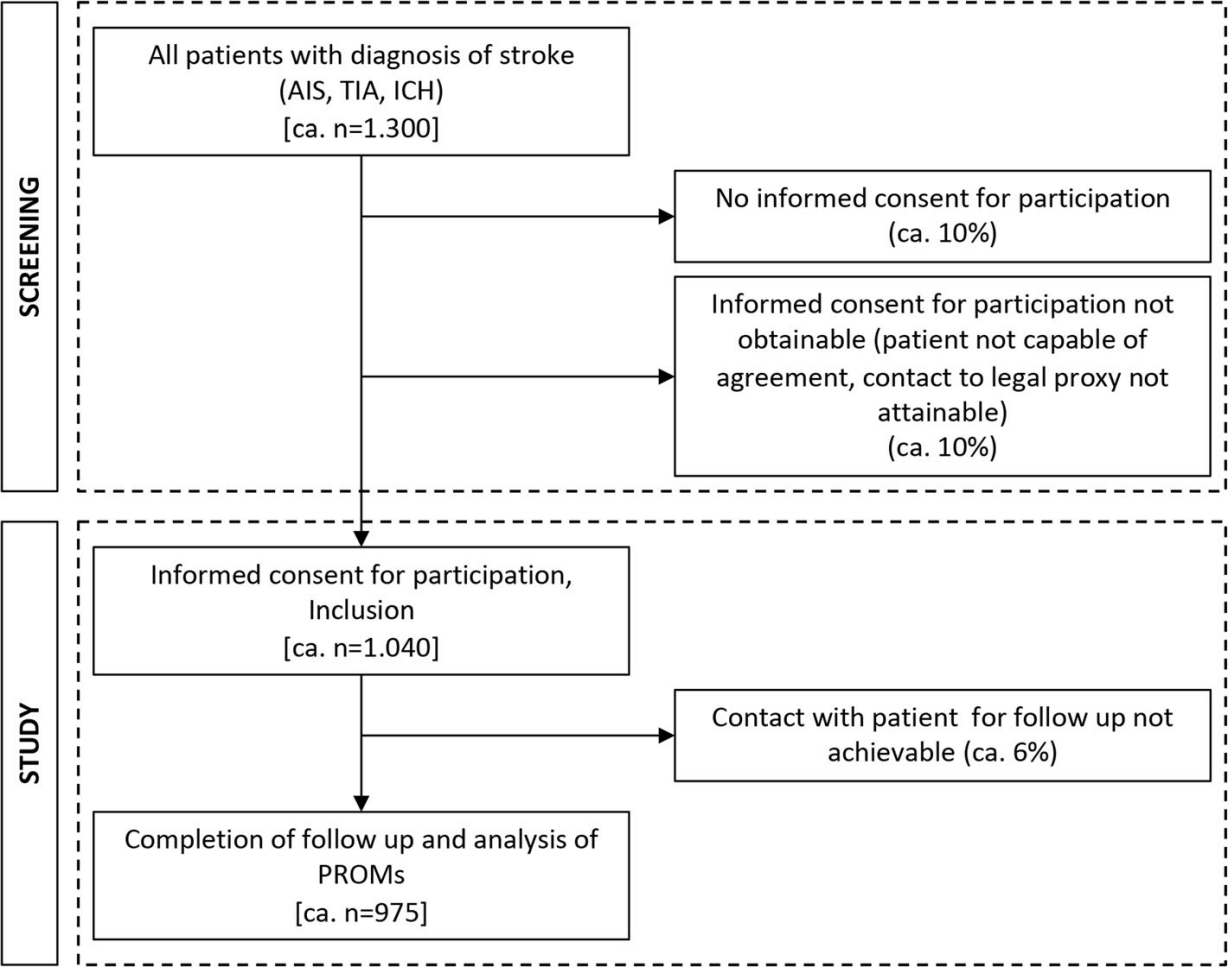
Estimated number of included patients after recruitment. Abbreviations: *AIS* acute ischemic stroke, *TIA* transient ischemic attack, *ICH* intracerebral hemorrhage

In the past years, approximately 1300 patients with a relevant diagnosis were admitted at the stroke unit of our hospital annually. Of these patients, 60% had an ischemic stroke, 32% had a transient ischemic attack, and 8% were admitted with intracerebral hemorrhage. With an expected dropout of approximately 25%, we aim to include *n* = 1040 patients (Fig. 1). This number allows for exploratory descriptive and inferential analyses with high precision and power, including subgroup analyses and the use of multivariate and longitudinal models. While the planned analyses are of exploratory nature, sample size calculations, done with GPower [7] show that in order to identify a small effect (H0 ρ^2^ = 0.01 and H1 ρ^2^ = 0.05 in a two-sided test with a power of 0.90, a regression analysis with 25 predictors the analysis of data of 846 cases are necessary, which is below the expected sample size.

### Eligibility criteria

Inpatients of the stroke unit will be screened for participation via the electronic health record (EHR) by study nurses after admission. They will not be involved in treatment of the patients and use set ups of the EHR to filter diagnoses and to obtain an overview of included and not included patients for minimizing losses.

### Arms and intervention

In order to implement the ICHOM-SSS into the clinical routine, it will be integrated into the medical center’s EHR to ease identification, inclusion, and storage of data. A process evaluation will be conducted, measuring the degree of feasibility in this study, acceptance of patients, clinical and administrative staff, practicability, realization, and adoption of the implementation process and execution of this assessment, reach, and fidelity to protocol (Table 1). These outcomes are chosen by means of the current standards of feasibility studies [6, 11, 18]. According to a mixed method approach, the evaluation is subdivided into quantitative and qualitative analyses. The quantitative part consists of two data quality and plausibility checks. The first analysis is conducted shortly after start of data collection, and the second is conducted after the data collection is completed.

**Table 1:** Quantitative and qualitative feasibility assessment; type and description of analysis

Domain	Operationalisation
Acceptance	Satisfaction with the intervention and its implementation
Practicability	Relevance of the intervention and compatibility with the specific setting
Realisation and adoption	Realisation: intend and action to employ the intervention Adoption: adjusted execution of the intervention to fit the setting and recording of these adjustments
Accessibility	Penetration of intervention and access for all designated and eligible recipients
Fidelity to protocol	Quality and of intervention delivery and adherence to implementation protocol

The qualitative data are collected in semi-structured interviews with patients as well as clinical and study staff. Aim of the interviews is to capture opinion and experience of the intervieews regarding the extended health evaluation with the focus on acceptance, adequacy, and purpose as well as facilitators and barriers of the implementation process. It is planned to conduct 15 patient interviews, matched for severity of stroke impact, measured with the simplified modified Ranking Scale questionnaire (smRSq). While the patient interviews will be held by phone after the 12 months follow up is completed, the staff interviews (*n* = 5) will be conducted twice; once shortly after the introduction of the ICHOM-SSS to the clinical routine and once after the termination of the enquiry phase. The first data check and staff interview serve not only as an inspection of feasibility but allow for potentially necessary adjustments to the process. The second data check and expert interview repeat and finalize the inspection of feasibility, respectively. Interviewing professionals of different contexts and patients shall increase validity of the results by capturing various perspectives on the enquiry.

If meeting the inclusion criteria, patients will be interviewed and their clinical records analyzed during inpatient stay concerning demographics, risk factors, and patient characteristics. The interview takes place twice; once as soon as possible after intake and the second at discharge. If attending the interviews or answering the questionnaire is not possible due to disability, e.g. aphasia, the patient’s proxy will be contacted and asked to provide the information.

**Fig. 2:**
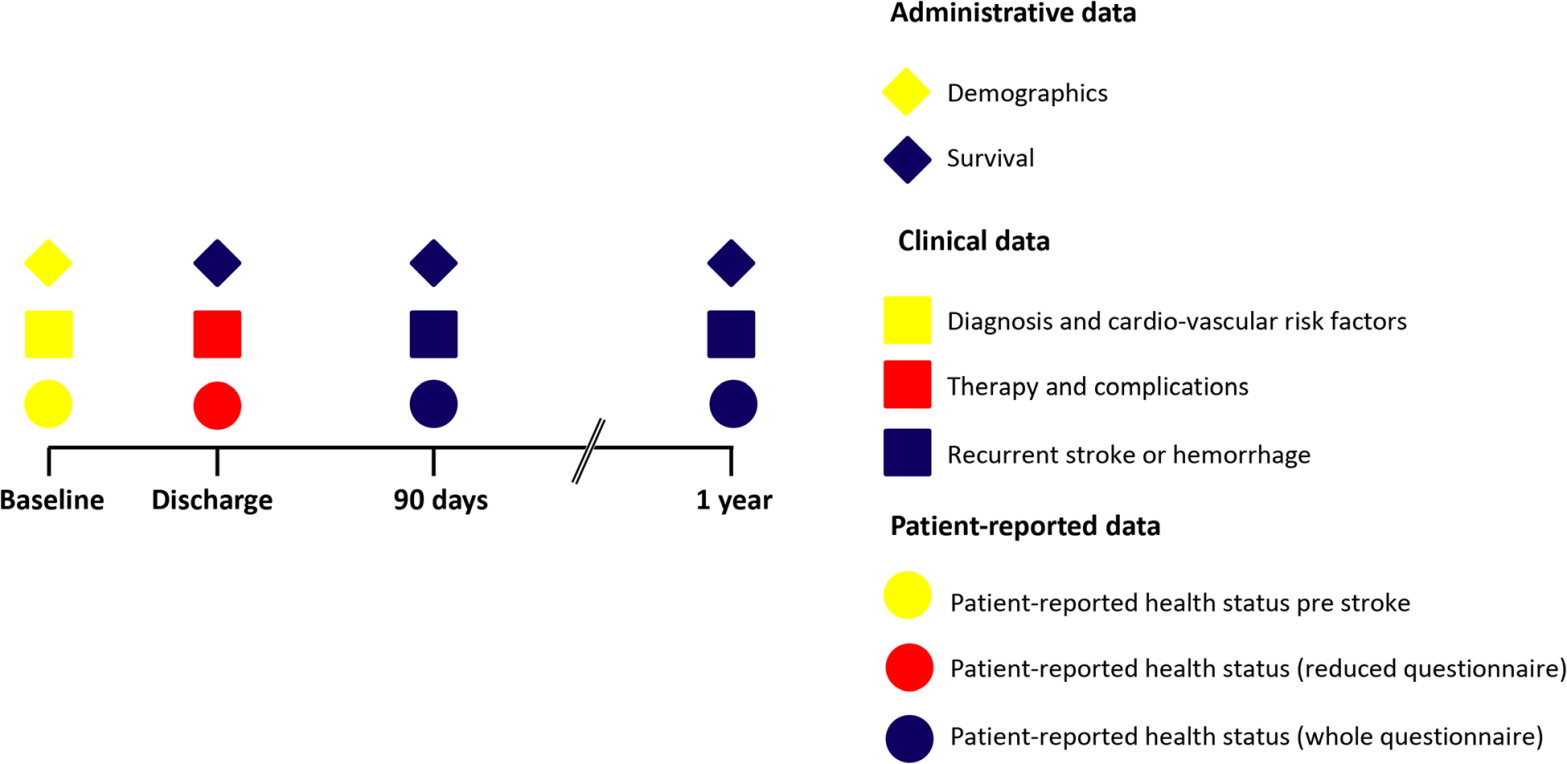
Timepoints and content of determined data

Assessment and parameters are collected according to the ICHOM-SSS(International Consortium for Health Outcomes Measurement (ICHOM), 2017), consisting of living situation, pre- and post-stroke functional status, cardiovascular risk factors including atrial fibrillation, the National Institute of Health Stroke Scale (NIHSS), and administrative data i.e. length of stay, survival, planned rehabilitation procedures, and discharge destination (Fig. 2). Follow up assessments take place 3 and 12 months after admission respectively. The assessment strategy is twofold. Firstly, a paper-and-pencil questionnaire is sent to the patients, consisting of a repetition of questions listed above, additional to the PROMs described below. Secondly, a short telephone interview takes place to assess the smRSq as well as record PROMs if patients were unable to fill in and send back the questionnaire themselves. If contact cannot be established postal or via three phone calls within 2 weeks patients will be excluded from further analysis.

### Outcome measures

The primary outcome of this project is the change in patient-reported global health after stroke over the course of 1 year. This is measured three and 12 months after stroke using the Patient-reported Outcomes Measurement Information System 10-Question Short Form (PROMIS-10) questionnaire. The PROMIS-10 has been shown to be feasible and valuable in the assessment of patients after stroke [22]. Relevant domains are physical and mental health, for both used a t-score calculated from a sum-score with standardized t-values, with M ± SD = 50 ± 10, as fit in healthy American subjects. Lower values will reflect a poorer outcome. Additional information may be gained from single questions concerning participation in social activity and general health. The direction of scoring is the same. Depressive and anxiety symptoms are assessed using the Patient Health Questionnaire-4 (PHQ-4). It is a four-item score and has been established as a self-reported written questionnaire in a broad German and American cohort containing different threshold values [19]. Its scale reaches from one to six for each subdomain, with higher scores indicating worse status, and a score above 3 considered positive for a potential presence of anxiety or depression. Lastly, according to the ICHOM-SSS, we will assess motor function by the smRSq. This is done by phone interview. The smRSq has been shown to reflect motor disability with a comparable value as the traditional mRS [4]. Its scale does not differ from the latter, and reaches from zero for no symptoms, to six for death.

### Data analysis and management

Research question 1: A process evaluation is performed to answer the first research question. This means process data on the implementation and enquiry process will be collected. Two structured analyses of process- and outcome data will be performed on congruency and completeness in order to detect potential discrepancies between conception and realization. The results of the evaluation will be examined via descriptive statistics. The interviews will be recorded, transcribed and analyzed using a realist thematic analysis approach [3], specifically a framework content analysis [9]. The thematic analysis approach is a method by which qualitative data is coded into themes. We will use a mainly deductive approach, as our feasibility outcomes are already predefined (see Table 1). Coding schemes are developed beforehand and discussed regularly. Nevertheless, we are open to the possibility of inductive theme generation, if data suggests. The results will be reported using consolidated criteria for reporting qualitative research [2].

Research questions 2 and 3: The data will be analysed by descriptive and inferential statistics, Depending on the outcome, (generalised) multilevel linear (patient-reported global health) and logistic (mental and motor health) regression analyses will be conducted. Factors that are believed to possibly influence the outcomes will be integrated in form of covariates. These will comprise demographics, pre-existing deficits and prior strokes, and deficits at admission (NIHSS) since recent studies have shown a negative influence of impaired motor function on different PROMIS domains(Katzan, Thompson, et al., 2018). Additionally, survival analyses (mortality) will be performed. Exploratory structural equation models are fitted to identify differential characteristic patterns of the outcomes. Further, exploratory subgroup analyses are planned, using regression models that fit the respective research questions. Results with *p* < .05 will be considered as statistically significant. As this study is of explorative nature, no adjustments for multiple testing will be made. However, the elevated risk of an occurrence of type-I errors will be regarded at the interpretation of the results. Missing values will be accounted for by using mixed modelling techniques. Sensitivity analyses will be performed when no mixed models are used. The influence of the NIHSS on the outcomes will be analysed separately using median and nonparametric tests.

#### Software

The electronic health record will be used for data collection and storage. For preparation and most quantitative data analyses, it is anticipated to use the software R (https://www.r-project.org) and IBM SPSS Statistics (https://www.ibm.com/analytics/spss-statistics-software). Lastly, the software MAXQDA (https://www.maxqda.com) will be used for qualitative data analyses.

#### Contacts

EPOS is conducted as a single-center study by the University Medical Center Hamburg-Eppendorf in collaboration of the Department of Neurology, Department of Medical Psychology, and the Office for Quality Management and Clinical Process Management. EPOS is funded by the Innovation Fund of the German Federal Joint Committee.

#### Perspective

Our study protocol represents the first prospective cohort study using the ICHOM Standard Set for Stroke to assess PROMs in stroke patients. Through open inclusion criteria, we attempt to gather a broad spectrum of stroke patients that is representative for the population of acute stroke patients treated on stroke unit in daily routine care in a German metropolitan regions. Information gathered from the process evaluation will help to understand the facilitators and barriers of the standard use of PROMs –also in the long term- in the routine stroke care. The analysis will show, what might have to be adjusted for a smooth and successful implementation and realization of an extended and long-term treatment assessment. Should the analysis show that such an implementation is feasible and accepted amongst affected persons, measures will be taken that the implemented enquiry remains in use within the routine health care at the stroke unit of the University Medical Centre Hamburg-Eppendorf after the end of the trial. Furthermore, the results of the process evaluation will help to accomplish and accelerate the implementation of such enquiries in other settings. Information analyzed from follow up assessments should describe health-related difficulties in everyday life and might show predictive patient or therapy-related factors concerning different groups of patients. Our analysis will help in identifying subgroups of stroke patients with increased risk of impaired quality of life after stroke that may be targeted by specific interventions. Overall, the information collected in our study will help to bring the patients’ perspective into the focus of stroke care and may inform future strategies for improving stroke treatment at different levels.

## Data Availability

The data will be deposited in a protected server of the University Medical Centre Hamburg-Eppendorf. Access is strongly regulated even for study personnel. Owing to the difficulty of de-identification (routine care, qualitative data, etc.), individual participant data will not be shared publicly. Upon reasonable request that includes a methodologically sound proposal for the usage of data that is also approved by the responsible review committee data may be shared.

## Abbreviations

AIS: Acute ischemic stroke
EHR: Electronic health record
ICD-10: International Statistical Classification of Diseases and Related Health Problems 10th revision
ICH: Intracerebral hemorrhage
ICHOM: The International Consortium for Health Outcomes Measurements
ICHOM-SSS: The International Consortium for Health Outcomes Measurements -Standard Set for Stroke
PHQ-4: Patient Health Questionnaire-4
PROMIS-10: Patient-reported Outcomes Measurement Information System 10-Question Short Form
PROMs: Patient-reported Outcome measures
smRSq: simplified modified Ranking Scale questionnaire
TIA: Transient ischemic attack
